# *CYP2J3 *Gene Delivery Reduces Insulin Resistance via Upregulation of eNOS in Fructose-treated Rats

**DOI:** 10.1186/1475-2840-10-114

**Published:** 2011-12-21

**Authors:** Xizhen Xu, Ling Tu, Luyun Wang, Xiaosai Fang, Dao Wen Wang

**Affiliations:** 1 Department of Internal Medicine and The Institute of Hypertension, Tongji Hospital, Tongji Medical College, Huazhong University of Science and Technology, Wuhan 430030, People's Republic of China; 2 Department of Emergency, Sun Yat-sen Memorial Hospital, Sun Yat-sen University, Guangzhou 510120, People's Republic of China

## Abstract

Accumulating evidence suggests that cytochrome P450 (CYP) epoxygenases metabolize arachidonic acid into epoxyeicosatrienoic acids (EETs) which play important roles in various pathophysiological processes. Interestingly, CYP-derived eicosanoids are vasodilatory, at least in part through their ability to activate eNOS and subsequent NO release. This study investigated the roles of eNOS in *CYP2J3 *gene delivery reducing blood pressure and improving insulin resistance in fructose-treated rats. *CYP2J3 *overexpression *in vivo *increased EET generation, reduced blood pressure and reversed insulin resistance as determined by insulin resistance index (HOMA-IR). Furthermore, administration of eNOS inhibitor L-NMMA significantly and partially abolished the beneficial effects of *CYP2J3 *gene delivery on hypertension and insulin resistance induced by fructose intake, and possible mechanism is associated with increased ET-1, ET_A_-receptor mRNA expression and reduced sensitivity of insulin to peripheral tissues and organs characterized by reduced activity of IRS-1/PI3K/AKT and AMPK signalling pathways. These data provide direct evidence that CYP2J3-derived EETs may alleviate insulin resistance at least in part through upregulated eNOS expression.

## Introduction

In vascular endothelium, Nitric oxide (NO) is produced by a constitutively expressed enzyme known as endothelial nitric oxide synthase (eNOS), which converts L-arginine to L-citrulline[1]. A great amount of evidences implicate NO plays an important role in controlling vascular tone and modulating blood flow to organs [1]. Interestingly, experimental evidences suggest that NO is involved in the pathogenesis of insulin resistance and diabetes. eNOS activity and the production of NO are chronically impaired in type 2 diabetes[2]. Decreased eNOS activity has been observed in both aortic endothelium and cardiac tissue of fructose-treated rats[3], and furthermore exogenous insulin therapy appears to improve endothelial function in patients with type 2 diabetes[4]. Our previous data indicated that fructose intake induced increased blood pressure, insulin resistance and downregulation of eNOS expression, and eNOS overexpression significantly decreased fructose-induced hypertension and insulin resistance in rats[5]. Taken together, these data suggest that insulin resistance and diabetes are characterized partly by endothelial dysfunction and potentially by altered eNOS expression and NO production.

It is well known that cytochrome P450 (CYP) epoxygenases metabolized arachidonic acid into four different *cis*-epoxyeicosatrienoic acids (EETs): 5,6-, 8,9-, 11,12-, and 14,15-EET. Human P450 2J2 (CYP2J2) and its rat homolog CYP2J3 are predominant enzymes responsible for the oxidation of endogenous arachidonic acid pools in vascular endothelium, cardiac myocytes, pancreas, and other tissues where they exert regulatory effects in normal and pathophysiological processes[6].

Accumulating evidence suggests that EETs play crucial and diverse roles in cardiovascular homeostasis. CYP epoxygenases and EETs upregulate eNOS expression and phosphorylation in bovine aortic endothelial cells via activation of Mitogen-activated protein kinase (MAPK), protein kinase C, and phosphatidylinositol 3-kinase (PI3K)/AKT signaling pathways[7,8]. Our previous study indicated that *CYP2J3 *overexpression significantly reduced insulin resistance, decreased blood pressure and prevented eNOS downregulation induced by fructose intake[9], and possibly *CYP2J3 *gene delivery reduced blood pressure through upregulated eNOS expression and downregulated endothelin-1 (ET-1) and endothelin receptor A (ETA) expression. Furthermore, *CYP2J3 *overexpression significantly improved insulin resistance, at least in part through eNOS, IRS-1, and PI3K/AKT signaling pathways, as well as adenosine monophosphate-activated protein kinase (AMPK) signaling pathways in liver, muscle, heart, and kidney[9]. Interestingly, CYP-derived eicosanoids are vasodilatory, at least in part through their ability to activate eNOS and subsequent NO release[10], however, whether *CYP2J3 *overexpression can decrease blood pressure and reduce insulin resistance induced by fructose via eNOS is still not completely clear.

In this study, we hypothesized that overexpression of *CYP2J3 *and the subsequent increase in production of EETs might attenuate hypertension and insulin resistance via upregulation of eNOS. Thus, the present study investigated the roles of eNOS in *CYP2J3 *gene delivery reducing blood pressure and improving insulin resistance in fructose-treated rats.

## Research design and methods

### Materials

Polyclonal antibodies to β-actin, PI3K, AKT, phosphorylated AKT (P-AKT), AMPK, phosphorylated AMPK (P-AMPK), insulin receptor substrate-1 (IRS-1), phosphorylated IRS-1, and eNOS were from Santa Cruz Biotechnology Inc. (Santa Cruz, CA); goat anti-rabbit, goat anti-mouse and rabbit anti goat horseradish peroxidase-conjugated secondary antibody were obtained from Sigma-Aldrich (St. Louis, MO); Enhanced chemiluminescence substrate (SuperSignal Substrate) was product of PIERCE (Rockford, IL); Plasmid purification kits from GIBCO (Grand Island, NY); Fructose, glucose, triglyceride and cholesterol reagents were purchased from Ningbo Chicheng Biocompany (Ningbo, China). 14,15-DHET ELISA Kit from Detroit R&D Inc. NG-Monomethyl-L-Arginine (L-NMMA) was from Sigma-Aldrich (St. Louis, MO); All other chemicals and reagents were purchased from Sigma-Aldrich unless otherwise specified. A full-length of CYP2J3 cDNA were cloned from rat liver RNA and then subcloned into plasmid of pcDNA3.1 in sense (p2J3(+)), and pcDNA-CYP2J3(+) was a generous gift from Dr. Zeldin (National Institute of Environmental Health Sciences, National Institutes of Health), specifically, we have re-sequenced it, and the results indicated that the sequence was correct.

### Animals

All animal experimental protocols were approved by The Academy of Sciences of China and complied with standards stated in the NIH Guidelines for the Care and Use of Laboratory Animals. Male Sprague-Dawley rats weighing 180 ± 20 g were obtained from the Experimental Animal Center of Shanghai (Shanghai, PRC). Experimental animals were housed at 25°C with 12 hour light/dark cycles and a relative humidity of 50% and allowed free access to normal chow and water throughout the study period. Animals were randomly assigned to different treatment groups and subjected to a one-week adaptation period.

### Fructose feeding protocol and gene delivery

Rats were randomly assigned to different treatment groups and subjected to a one-week adaptation period for systolic blood pressure measurement via the tail-cuff method as described below. Following this (i.e. beginning at week 0), rats were fed normal rat chow and either water (n = 10) with or without containing 10% fructose (n = 40) for a total of 6 weeks. Systolic blood pressure was measured weekly until week 6, and gene delivery protocols were undertaken at week 3 as described previously[9]. Briefly, 3 weeks after fructose feeding, rats were anesthetised with ethylether and received sublingual vein injections of various vectors dissolved in 0.9% NaCl (at 1 mg/ml) at a dose of 5 mg/kg body weight. Normal-treated rats received injection of 0.9% NaCl (Normal). Fructose-treated rats were further divided into four groups: Fructose (fructose+pcDNA3.1); Fructose+L-NMMA (fructose+pcDNA3.1+L-NMMA; L-NMMA (100mg/Kg.d) was given by gavage from the beginning of fructose drinking to the end of the study); Fructose+pcDNA-CYP2J3(+) (received injection of pcDNA-CYP2J3(+)); Fructose+pcDNA-CYP2J3(+)+L-NMMA (n = 8 for each group).

### Blood pressure measurement

Blood pressure was measured as described previously[9]. In brief, systolic blood pressure was measured weekly in conscious rats with a manometer-tachometer (Rat Tail NIBP System, ADI Instruments, Australia) using the tail-cuff method. Rats were placed in a plastic holder mounted on a thermostatically controlled warm plate that was maintained at 35°C during measurements. An average value from 5 blood pressure readings (that differed by no more than 2 mmHg) was determined for each animal after they became acclimated to the environment. All blood pressure measurements were made between 09:00 h and 12:00 h.

### Serum and urine analysis

Just prior to gene delivery at week 3 of the study, approximately 0.5 ml of blood was drawn from the tail vein of each rat. After coagulation, serum was collected by centrifugation and stored at -80°C. Urine samples were collected over a 24-hour period prior to gene delivery as described previously[9]. Three weeks after gene delivery (i.e. at week 6 of the study), serum and urine samples were collected and analyzed in a similar manner from 10 rats from each of the above groups. Serum glucose, serum cholesterol, serum triglyceride, Serum insulin levels, serum LDL-C, serum HDL-C, and urinary osmolarity were measured as described before[9], and insulin resistance was calculated using the homeostasis model assessment (HOMA-IR = fasting glucose × fasting insulin/22.5) method. We measured serum nitric oxide (NO) concentrations using the Griess method.

### Evaluation of plasma and urine 14,15-DHET by ELISA

To assess *in vivo *EET production, an ELISA kit (Detroit R&D) was used to determine concentrations of the stable EET metabolite 14,15-dihydroxyeicosatrienoic acid (14,15-DHET) in the plasma and urine of rats. Briefly, eicosanoids were extracted from the plasma and urine samples three times with ethyl acetate after acidification with acetic acid (to convert EETs into DHETs). After evaporation, saponification with 0.4N KOH in methanol and re-extraction, 14,15-DHET was dissolved in 30 μl DMF and quantified by ELISA according to the manufacturer's instructions as previously described[9].

### Western blot analysis

Three weeks after gene injection, rats from each group were anesthetised with pentobarbital (100 mg/kg i.p.) and skeletal muscles, aortas, hearts, kidneys and livers were excised, frozen in liquid nitrogen and stored at -80°C. Related protein expression was determined by western blot as described previously[9]. In brief, tissue proteins were extracted using TRIZOL reagent and protein concentrations were estimated by the Bradford method. Twenty micrograms of protein per lane were separated with 10% SDS/PAGE gels and electrophoretically transferred onto PVDF membranes. Membranes were then probed using various antibodies and the ECL system was used to visualize the separated proteins. Blots were stripped and reprobed with β-actin as a loading control.

### Statistical analysis

Continuous data were expressed as means ± S.E.M. Comparisons between groups were performed by a one-way analysis of ANOVA with *post hoc *analyses performed using the Student-Newman-Keuls method. Statistical significance was defined as *P *< 0.05.

## Results

### Metabolic parameters changes in fructose-treated rats

All rats in the study were assessed for a variety of physiological parameters 3 weeks after receiving either control or fructose-containing drinking water. As expected, consumption of fructose-containing water resulted in significantly increased levels of serum insulin and serum triglyceride, and significantly decreased urine osmolarity (all *P *< 0.05) (Table 1). Insulin resistance (HOMA-IR) was also significantly increased in fructose-treated rats (*P *< 0.05). These data indicate that fructose administration induced insulin resistance, and hypoosmolar diuresis as described previously[11].

**Table 1:** Physiological parameters determined in rats after 3 weeks of administration of control or fructose-containing drinking water

Variable	Control Group (n = 10)	Fructose-treated Group (n = 40)
Glucose (mmol/l)	4.29 ± 0.2	5.22 ± 0.23
Insulin (mIU/l)	9.51 ± 0.39	18.21 ± 1.99**
HOMA-IR	1.71 ± 0.56	4.14 ± 2.69**
Triglyceride (mmol/l)	0.47 ± 0.06	1.06 ± 0.08*
Urine osmolarity (mOsml/Kg H_2_O)	826 ± 110	427 ± 33.69**
Body weight (g)	212 ± 36	334.18 ± 56.74

Values shown are mean ± SEM. *P < 0.05 vs. control group; **P < 0.001 vs. control group.

### Effects of CYP2J3 gene delivery on CYP2J3 protein expression

**Figure 1:**
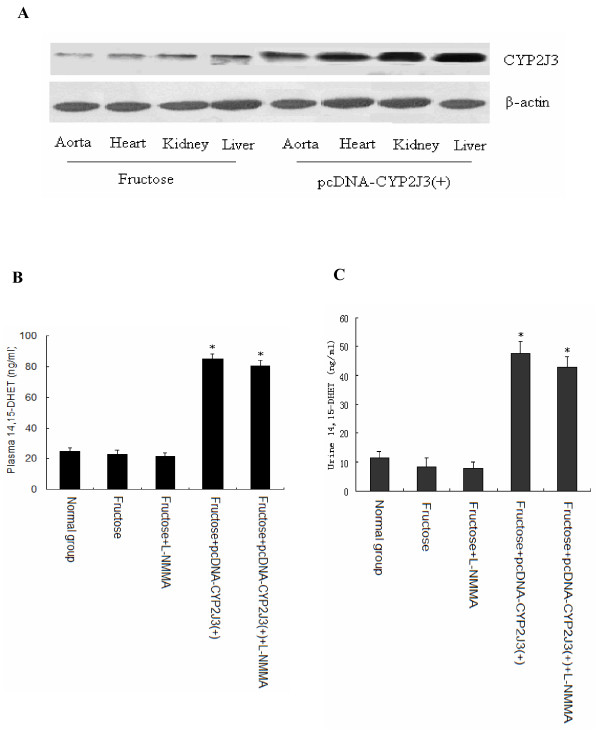
**Effects of *CYP2J3 *gene delivery on CYP2J3 protein expression, plasma and urinary 14,15-DHET levels**. A, CYP2J3 protein levels were increased in aorta, heart, liver and kidney of fructose-treated rats 3 weeks following injection of CYP2J3(+).B, Plasma 14,15-DHET levels were increased in fructose-treated rats injected with CYP2J3(+) compared to rats in Fructose group. C, Urinary 14,15-DHET levels were increased in fructose-treated rats injected with CYP2J3(+) compared to rats in Fructose group. *P < 0.05 vs. normal group or fructose group; n = 10 per group. Values shown are mean ± SEM from each group of rats, respectively.

There weeks after gene delivery (i.e. at week 6 of the study), CYP2J3 protein levels were increased in the aorta, heart, liver and kidney of fructose-treated CYP2J3(+) (F+CYP2J3(+)) rats compared to fructose-treated rats (Figure 1A). Importantly, CYP2J3 functionality was demonstrated by the nearly 4-fold increase in plasma and urinary 14,15-DHET levels in rats injected with CYP2J3(+) compared to those in normal group (Figure 1B and 1C). Administration with L-NMMA had no significant effects on plasma and urinary 14,15-DHET levels as shown in Figure 1B and 1C. These data indicate that CYP2J3 was successfully and efficiently expressed in experimental animals.

### Effects of L-NMMA administration on fructose-induced hypertension and pathophysiological changes in rats

**Figure 2:**
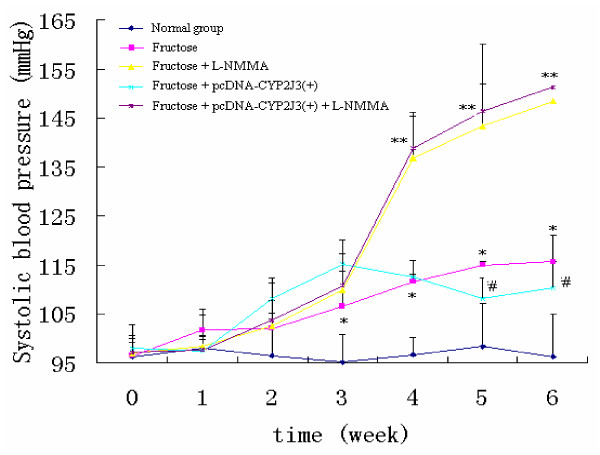
**Effects of L-NMMA on fructose-induced hypertension with *CYP2J3 *gene delivery in rats**. The elevation of systolic blood pressure observed in fructose-treated rats was decreased 1, 2 and 3 weeks after injection of CYP2J3(+) (at weeks 4, 5 and 6 of the study). Administration of L-NMMA significantly increased blood pressure in rats of Fructose group. Interestingly, administration of L-NMMA significantly increased blood pressure in rats of Fructose+pcDNA-2J3(+) group. Values shown are mean ± SEM from each group of rats, respectively.*P < 0.05 compared to rats treated without fructose, ^#^*P *< 0.05 compared to rats treated with fructose, **P < 0.05 compared to rats in Fructose + pcDNA-CYP2J3(+) group; n = 4 to 10 per group per time point.

As expected, consumption of fructose-containing water resulted in significantly increased levels of blood pressure, and injection of CYP2J3 to fructose-treated rats resulted in decreased systolic blood pressure 2 and 3 weeks after injection (i.e. at weeks 5 and 6 of the study) compared to that observed in fructose-treated rats (Figure 2). Administration of L-NMMA to fructose-treated rats resulted in increased systolic blood pressure compared to that observed in fructose-treated rats (Figure 2) (*P *< 0.05). Interestingly, administration of L-NMMA significantly prevented the decrease in blood pressure levels induced by CYP2J3 overexpression in fructose-treated rats (Figure 2) (*P *< 0.05). These data indicate that *CYP2J3 *gene delivery significantly decreased systolic blood pressure in fructose-treated rats at least in part through eNOS related signal pathways.

Other physiological and biochemical parameters related to hyperinsulinemia were assessed in 10 rats per experimental group 3 weeks after gene delivery (week 6) (Table 2). Compared to values in the normal group, serum insulin, insulin resistance (HOMA-IR) and serum triglycerides were all higher, while urine osmolarity was lower in fructose-treated rats (all *P *< 0.05) (Table 2). With the exception of serum triglyceride levels, all of these changes were prevented by *CYP2J3 *gene delivery in rats with fructose-treated (Table 2). Interestingly, administration of L-NMMA significantly and partially reversed the effects of *CYP2J3 *gene delivery on serum insulin, insulin resistance (HOMA-IR) and urine osmolarity in fructose-treated rats (all *P *< 0.05) (Table 2). These data indicate that CYP2J3 overexpression markedly attenuated fructose-induced insulin resistance in rats at least in part through eNOS related signal pathways.

**Table 2:** Physiological parameters determined in rats 3 weeks following injection of pcDNA3.1 vector or pcDNA-CYP2J3(+)

	Treatment Group				
	**Normal Water-treated**	**Fructose-treated**			
**Variable**	**Normal**	**Fructose**	**Fructose+L-NMMA**	**Fructose+ pcDNA-CYP2J3(+)**	**Fructose+ pcDNA-CYP2J3(+)+L-NMMA**

Glucose (mmol/L)	4.56 ± 0.68	5.04 ± 0.62	6.43 ± 1.09	5.84 ± 0.42	6.55 ± 0.84
Insulin (mIU/L)	9.65 ± 0.83	23.84 ± 1.37*	25.41 ± 3.25	6.24 ± 0.33^#^	22.54 ± 0.99**
HOMA-IR	1.85 ± 0.32	5.34 ± 0.38*	7.26 ± 0.56	1.62 ± 0.42^#^	6.56 ± 0.28**
Triglyceride (mmol/L)	0.56 ± 0.32	1.23 ± 0.17*	1.06 ± 0.17	1.19 ± 0.24^#^	1.14 ± 0.28
Urine osmolarity (mOsml/Kg H_2_O)	857 ± 101	377.4 ± 69.95*	346.14 ± 34.24	763 ± 54.49^#^	258 ± 48.68**
Body weight (g)	273 ± 30	356 ± 29.3	347 ± 35	332 ± 19.7	379 ± 20

Values shown are mean ± SEM. *P < 0.05 vs. Normal group; ^#^*P *< 0.05 vs. Fructose group; **P < 0.05 vs. Fructose+ pcDNA-CYP2J3(+) group.

### Effects of L-NMMA administration on eNOS expression and activity in rats

**Figure 3:**
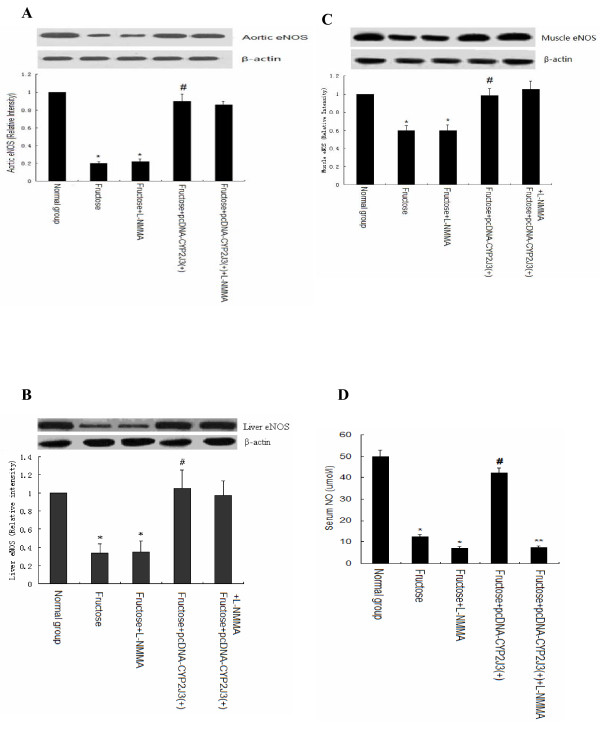
**Effects of L-NMMA administration on eNOS expression and activity**. Levels of eNOS expression in aorta (A), liver (B) and muscle (C) were assessed by western blot at week 6 of the study. Serum NO levels (D) were determined by Griess method at week 6 of the study. Representative western blot of eNOS and corresponding densitometric quantification of three experiments are shown. Values shown are mean ± SEM. *P < 0.05 vs. Normal group; ^#^*P *< 0.05 vs. Fructose group; **P < 0.05 vs. Fructose+ pcDNA-CYP2J3(+) group.

As expected, compared to expression in corresponding control animals, eNOS protein expression was down-regulated in aorta, liver and skeletal muscle in fructose-treated rats (Figure 3A, 3B and 3C), and this effect was not observed in fructose-treated rats injected with *CYP2J3 *(Figure 3A, 3B and 3C). Interestingly, administration of L-NMMA didn't affect eNOS expression in aorta, liver and skeletal muscle in fructose-treated rats (Figure 3A, 3B and 3C). Importantly, fructose intake markedly reduced serum NO levels, and administration of L-NMMA further decreased serum NO levels as shown in Figure 3D. Interestingly, *CYP2J3 *gene delivery significantly reversed the changes in NO levels induced by fructose, and administration of L-NMMA significantly and completely abolished the beneficial effects of *CYP2J3 *gene delivery on NO levels (Figure 3D). These data suggest that administration of L-NMMA blocked eNOS activity, and didn't affect eNOS expression.

### Effects of L-NMMA administration on intracellular signaling pathways in rats

To investigate potential mechanisms underlying the observed effects of L-NMMA administration on improved insulin resistance by *CYP2J3 *gene delivery in fructose-treated rats. The protein expression levels of a variety of intracellular signaling pathway molecules were investigated in liver and skeletal muscle of rats in all treatment groups 3 weeks following gene delivery. The related signaling molecules that were assessed included phosphorylated IRS-1(IRS-1-Tyr(P)989 and IRS-1-Tyr(P)307), PI3K, P-AKT and P-AMPK.

As expected, phospho-Y989-IRS-1 levels were similarly decreased in liver and skeletal muscle, whereas phospho-S307-IRS-1 was significantly increased (data not shown). *CYP2J3 *gene delivery significantly reversed the changes in IRS-1 and phospho-IRS-1 levels induced by fructose both in liver and skeletal muscle (data not shown), and interestingly, administration of L-NMMA significantly partially abolished the beneficial effects of *CYP2J3 *gene delivery on IRS-1 and phospho-IRS-1 expression levels induced by fructose in rats.

**Figure 4:**
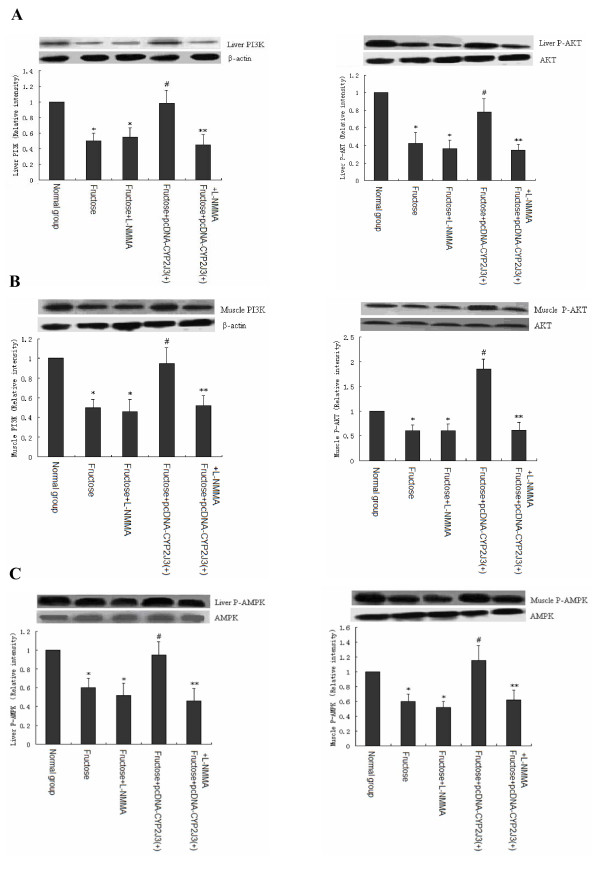
**Effects of *CYP2J3 *gene delivery on activation of rat insulin receptor signaling and AMPK phosphorylation**. Levels of, PI3K and P-AKT in liver (A) and muscle (B), and P-AMPK in liver and muscle (C) were assessed by western blot at week 6 of the study. Representative western blot of protein above and corresponding densitometric quantification of three experiments are shown. Values shown are mean ± SEM. *P < 0.05 vs. Normal group; ^#^*P *< 0.05 vs. Fructose group; **P < 0.05 vs. Fructose+ pcDNA-CYP2J3(+) group.

A similar pattern of expression was observed for all the molecules except IRS-1 and phospho-IRS-1 expression levels in all tissues that were examined. Specifically, as expected, tissue protein levels of the specified signaling molecules were significantly decreased in fructose-treated rats compared to levels in rats of normal group respectively. The only fructose-treated rats in which these decreases were not observed were those that were with *CYP2J3 *gene delivery. PI3K and P-AKT level data for rat liver and skeletal muscle are shown in Figure 4A and 4B, and for P-AMPK are shown in Figure 4C. *CYP2J3 *gene delivery reversed changes in protein expression and phosphorylation induced by fructose in rats. Interestingly, administration of L-NMMA significantly partially abolished the beneficial effects of *CYP2J3 *gene delivery on protein expression and phosphorylation induced by fructose in rats. These data indicated that *CYP2J3 *gene delivery significantly improved insulin resistance induced by fructose in rats at least in part through eNOS related signal pathways.

## Discussion

This study was undertaken to examine the roles of eNOS in *CYP2J3 *gene delivery reducing blood pressure and improving insulin resistance in fructose-induced insulin resistant rats. Results showed that a single intravenous injection of *CYP2J3 *in the eukaryotic expression plasmid pcDNA reduced blood pressure and improved sensitivity to insulin in peripheral tissues and organs in fructose-fed rats. Furthermore, administration of eNOS inhibitor L-NMMA significantly and partially abolished the beneficial effects of *CYP2J3 *gene delivery on hypertension and insulin resistance induced by fructose intake, and possible mechanism is associated with increased ET-1, ET_A_-receptor mRNA expression (data not shown) and reduced sensitivity of insulin to peripheral tissues and organs. These data provide direct evidence that CYP2J3-derived EETs may alleviate insulin resistance at least in part through upregulated eNOS expression.

Recent studies indicate that cytochrome P450 (CYP) epoxygenase decreased blood pressure in various hypertensive animal models. Overexpression of P450 epoxygenases attenuates the development of hypertension and improves cardiac function in spontaneous hypertensive rats [12]. CYP2J2 and CYP2C8 transgenic mice demonstrated lower mean arterial pressure during coadministration of N-nitro-l-arginine methyl ester and indomethacin[13]. In another experiment, a high-salt diet and subcutaneous angiotensin II was administered over 4 week. The angiotensin/high-salt-induced increase in systolic blood pressure, proteinuria, and glomerular injury was significantly attenuated in CYP2J2 and CYP2C8 transgenic mice compared to wild-type controls[13]. Interestingly, increases in plasma trans-EETs by the inhibition of soluble epoxide hydrolase markedly reduced blood pressure in spontaneously hypertensive rats[14]. In contrast, blood pressure increased significantly in female mice lacking cytochrome P450 2J5[15]. Overexpression of P450 epoxygenases resulted in increased aortic eNOS expression in vivo and in vitro, and its possible mechanisms were associated with the activation of MAPK and protein kinase C signaling pathways[7]. Interestingly, further study indicated that CYP epoxygenase metabolites EETs-induced up-regulation of eNOS phosphorylation and expression appears to involve in both PI3K/Akt and MAPK pathways [8]. CYP epoxygenase-derived eicosanoids are vasodilatory, largely through their ability to activate eNOS and NO release[16]. From the data described above, we can see that CYP epoxygenase and its metabolites EETs decrease blood pressure in some hypertensive animal models at least in part through increased eNOS expression and activity. Our recent data suggest that *CYP2J3 *gene delivery significantly reduced blood pressure in fructose-treated rats, however, whether *CYP2J3 *gene delivery reduces blood pressure partially by the upregulation of eNOS remains to be further elucidated. In this study, we found that administration of eNOS inhibitor L-NMMA significantly and partially abolished the beneficial effects of *CYP2J3 *gene delivery on hypertension induced by fructose intake, and its possible mechanism is associated with increased ET-1, ET_A_-receptor mRNA expression (data not shown). These data demonstrated that *CYP2J3 *gene delivery significantly reduced blood pressure at least in part through the upregulation of eNOS in fructose-treated rats.

Previous experimental evidence suggests that NO is involved in the pathogenesis of insulin resistance and diabetes[17,18]. Molecular genetic study demonstrated that CYP2J2 G-50T polymorphism may contribute to the pathogenesis of type 2 diabetes, partially by effects on insulin resistance, in patients with younger onset type 2 diabetes[19]. Furthermore, Recent data indicate that reduced NO-cGMP signaling contributes to vascular inflammation and insulin resistance induced by high-fat feeding[20]. In contrast, increased eNOS expression reduces hyperinsulinemia and improves insulin resistance in fructose-treated rats[5]. Our recent data suggest that *CYP2J3 *gene delivery significantly improved insulin resistance in fructose-treated rats, however, whether *CYP2J3 *gene delivery improves insulin resistance partially by the upregulation of eNOS remains to be further elucidated. Previous data demonstrate that eNOS overexpression activates the IRS-1/PI3K/AKT signalling pathways, which indicate that eNOS overexpression activates insulin/insulin receptor-related signaling pathways and suggest that eNOS may potentiate insulin receptor signaling in muscles and thus improve insulin sensitivity [5]. Small molecule-mediated activation of AMPK improves insulin resistance in ob/ob mice [21] and represents a promising approach for the treatment of type 2 diabetes and the metabolic syndrome [22]. Interestingly, eNOS overexpression significantly increased AMPK phosphorylation in fructose-treated rats[5]. In this study, we found that administration of eNOS inhibitor L-NMMA significantly and partially abolished the beneficial effects of *CYP2J3 *gene delivery on insulin resistance induced by fructose intake, which is supported by the results of recent study that increased NO availability attenuates high fat diet induced metabolic alterations and gene expression associated with insulin resistance[23], and its possible mechanism is associated with inhibited IRS-1/PI3K/AKT and AMPK signalling pathways. These data demonstrated that *CYP2J3 *gene delivery significantly improved insulin resistance at least in part through the upregulation of eNOS in fructose-treated rats.

In conclusion, we have demonstrated that *CYP2J3 *gene delivery significantly reduced hypertension and improved insulin resistance at least in part through the upregulation of eNOS expression in fructose-treated rats. These effects were associated with decreased ET-1 and ET_A _mRNA expression in aorta and activation of the IRS-1/PI3K/AKT and AMPK signaling pathways in liver and muscle. The ability of CYP epoxygenase delivery to exert a broad spectrum of beneficial effects is mainly attributed to the activation of eNOS related signalling pathways, and warrants further investigation of this approach in the treatment of hypertension associated with insulin resistance and diabetes in humans.

## Competing interests

The authors declare that they have no competing interests.

## Authors' contributions

XZ and DWW designed the study, analyzed the data and wrote the manuscript; XZ and LW carried out the experiments; LT and XF edited and revised the manuscript; All authors read and approved the final manuscript.
