# Correction: CYP2J3 gene delivery reduces insulin resistance via upregulation of eNOS in fructose-treated rats

**DOI:** 10.1186/1475-2840-13-17

**Published:** 2014-02-11

**Authors:** Xizhen Xu, Ling Tu, Luyun Wang, Xiaosai Fang, Dao Wen Wang

**Affiliations:** 1 Department of Internal Medicine and The Institute of Hypertension, Tongji Hospital, Tongji Medical College, Huazhong University of Science and Technology, Wuhan 430030, People’s Republic of China; 2 Department of Emergency, Sun Yat-sen Memorial Hospital, Sun Yat-sen University, Guangzhou 510120, People’s Republic of China

## Correction

We deeply regret that we have inadvertently made a minor mistake in our publication [1] and we feel it necessary to correct this error. Following publication of our work, we reviewed all the Western blots of the experiments; all showed an identical trend in terms of scientific interpretation. Unfortunately, it was subsequently noticed that Figure one A (Figure 1 here) (CYP2J3 expression) was not the original unedited (un-cropped) blot, and we would like to correct that inadvertent error. The following Western blot (Figure 1) therefore replaces Figure one A in the original article. We recognize that this Western blot is not as clean, but it clearly presents the original without modifications (cropping) and thus can avoid confusion for the readers. We wish to emphasize that the experiments on CYP2J3 overexpression with Western blot analysis were repeated 4 times, with identical results obtained each time. The error had no effect on the scientific content or conclusions. We deeply apologize that this occurred, and take responsibility for the error and hereby present the correction.

**Figure 1:**
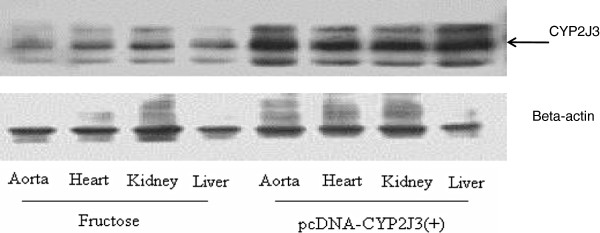
**Effects of *CYP2J3 *gene delivery on CYP2J3 protein expression, plasma and urinary 14,15-DHET levels.** CYP2J3 protein levels were increased in aorta, heart, liver and kidney of fructose-treated rats 3 weeks following injection of CYP2J3(+).
